# Isoflurane conditioning improves functional outcomes after peripheral nerve injury in a sciatic cut repair murine model

**DOI:** 10.3389/fneur.2024.1406463

**Published:** 2024-08-15

**Authors:** Yameng Xu, Ying Yan, Gregory J. Zipfel, Matthew MacEwan, Wilson Z. Ray, Umeshkumar Athiraman

**Affiliations:** ^1^The Institute of Materials Science & Engineering, Washington University, St. Louis, MO, United States; ^2^Department of Neurological Surgery, Washington University, St. Louis, MO, United States; ^3^Department of Neurology, Washington University, St. Louis, MO, United States; ^4^Department of Orthopedic Surgery, Washington University, St. Louis, MO, United States; ^5^Department of Biomedical Engineering, Washington University, St. Louis, MO, United States; ^6^Department of Anesthesiology, Washington University, St. Louis, MO, United States

**Keywords:** isoflurane conditioning, neuroprotection, peripheral nerve injury, functional outcomes, myelin regeneration, axonal regeneration

## Abstract

**Introduction:**

Anesthetic conditioning has been shown to provide neuroprotection in several neurological disorders. Whether anesthetic conditioning provides protection against peripheral nerve injuries remains unknown. The aim of our current study is to investigate the impact of isoflurane conditioning on the functional outcomes after peripheral nerve injury (PNI) in a rodent sciatic nerve injury model.

**Methods:**

Adult male Lewis rats underwent sciatic nerve cut and repair and exposed to none (Group 1, sham), single isoflurane exposure (Group 2), three-time isoflurane exposure (Group 3), and six-time isoflurane exposure (Group 4). Isoflurane conditioning was established by administration of 2% isoflurane for 1 hour, beginning 1-hour post sciatic nerve cut and repair. Groups 3 and 4 were exposed to isoflurane for 1 hour, 3 and 6 consecutive days respectively. Functional outcomes assessed included compound muscle action potential (CMAP), evoked muscle force (tetanic and specific tetanic force), wet muscle mass, and axonal counting.

**Results:**

We observed an increase in axons, myelin width and a decrease in G-ratio in the isoflurane conditioning groups (3- and 6-days). This correlated with a significant improvement in tetanic and specific tetanic forces, observed in both groups 3 and 4.

**Discussion:**

Isoflurane conditioning (3- and 6-day groups) resulted in improvement in functional outcomes at 12 weeks post peripheral nerve injury and repair in a murine model. Future experiments should be focused on identifying the therapeutic window of isoflurane conditioning and exploring the underlying molecular mechanisms responsible for isoflurane conditioning induced neuroprotection in PNI.

## Introduction

The incidence of peripheral nerve injuries (PNI) represents approximately 2–5% of all the trauma cases resulting, in healthcare expenditures of over $7 billion per year in the United States (1, 2). PNI represents a major health concern that broadly impacts patient’s functional status, emotional health, and quality of life (1, 2). Traumatic nerve injuries more commonly affect younger patients who are at their peak of economic productivity resulting in an immensely personal and family burden (3, 4). Though, significant advancements (microsurgical approaches) have been achieved in recent years in the surgical management of PNIs, functional recovery still remains poor (5, 6). Therefore, new treatment strategies are urgently needed to improve functional outcomes in patients following PNI.

Conditioning is the concept whereby the organ’s inherent resistance to injury can be enhanced by exposure to a sub-lethal injurious stimulus (7, 8). Conditioning strategies are powerful and remarkably pleiotropic, as it exerts protection on all major cell types including neurons, glia, and vascular cells (7, 8). Several distinct conditioning agents have been demonstrated to provide robust protection in a variety of organ systems through conditioning-based mechanisms (9–12). Several studies have implicated the protective role of commonly used volatile anesthetics in numerous brain injuries such as stroke, subarachnoid hemorrhage, intracerebral hemorrhage, traumatic brain injury, spinal cord injury and others (11, 13–16). However, data related to volatile anesthetics and neuroprotection in PNIs is lacking. The aim of our current proposal is to examine the impact of isoflurane conditioning on the functional outcomes following PNI, utilizing a rodent cut and repair model. Our hypothesis is that isoflurane conditioning will improve functional outcomes after PNI and repair.

## Methods

### Experimental design

**Figure 1:**
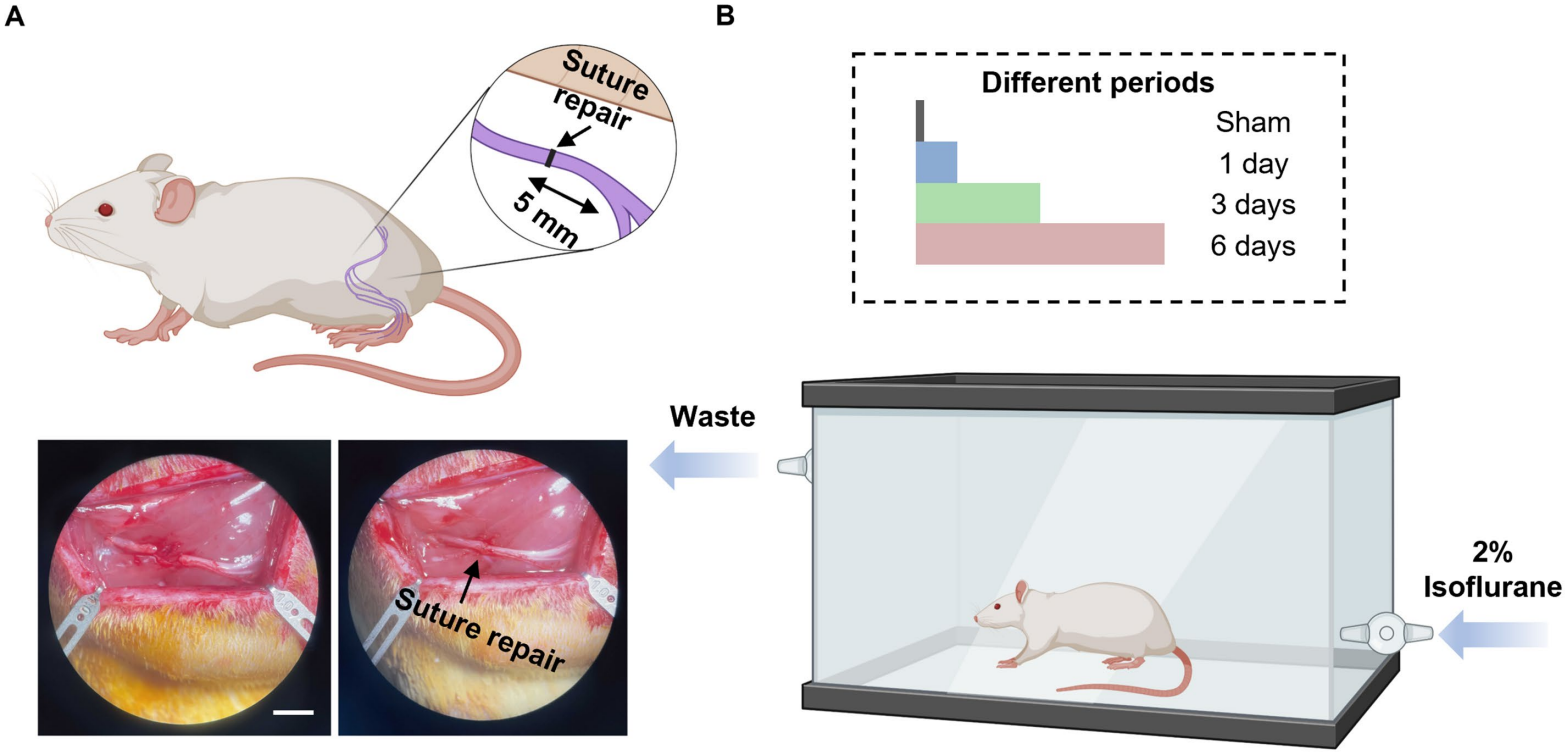
Diagram illustrations of study design. **(A)** Schematic diagram of nerve transection injury model and nerve repair (top), and bottom figure shows the representative images of nerve transection (left) and nerve repair (right). Scale bar, 5 mm. **(B)** Schematic diagram of isoflurane conditioning after the sciatic cut repair in rats with sham (no isoflurane exposure), 1-day, 3-days, and 6-days treatment with isoflurane. This figure was created using Biorender.com.

Thirty-two adult male Lewis rats (250 to 275 g, Charles River Laboratories, Wilmington, MA) underwent sciatic nerve transection and subsequent immediate repair. Eight rats were randomized into four groups (Table 1), which received either no exposure (Group I, sham group), 1 day (Group II), 3 days (Group III), or 6 days (Group IV) of 1-h of 2% isoflurane with oxygen as a carrier gas. At 12 weeks post-surgery, functional recovery was evaluated by measuring compound muscle action potentials (CMAPs) from the *tibialis anterior* (TA) muscle, evoked muscle force measurements from the *extensor digitorum longus* (EDL) muscle, and muscle mass of the EDL muscle. Immediate post functional recovery assessment, the sciatic nerves were harvested to analyze axonal counts and the microstructure of regenerated axons. The overall design of the study is represented in Figure 1 and Table 1.

**Table 1:** Study design.

Group	Group ID	Group size	Surgery model	Isoflurane conditioning
I	Sham	*N* = 8	Transection + repair	None (sham)
II	Isoflurane-postC1	*N* = 8	Transection + repair	2% isoflurane for 1 h, 1-h post-injury (1 day)
III	Isoflurane-postC3	*N* = 8	Transection + repair	2% isoflurane for 1 h, 1-h post-injury (3 days)
IV	Isoflurane-postC6	*N* = 8	Transection + repair	2% isoflurane for 1 h, 1-h post-injury (6 days)

### Surgical procedures

Surgical procedures and perioperative care measures were conducted in compliance with guidelines set by the Washington University Institutional Animal Use and Care Committee and the National Institutes of Health (Protocol # 21–0192). All animals were housed in a central animal care facility and provided with food (PicoLab rodent diet 20, Purina Mills Nutrition International, St. Louis, MO) and water *ad libitum*. Surgical tools were sterilized by ethylene oxide gas and all surgical procedures were performed under aseptic conditions. Anesthetic induction and maintenance were achieved by 4 and 2% isoflurane, respectively, for the sciatic cut repair surgery and for the end point assessments. A single dose of Buprenorphine SR (1.2 mg/kg, subcutaneous) was administered 1 h before surgery to alleviate postoperative pain and discomfort.

### Sciatic nerve cut repair model

A gluteal muscle incision was made to expose the right sciatic nerve and was freed from the connective tissue. The right sciatic nerve was then transected at 5 mm proximal to the sciatic trifurcation and reconstructed by securing the proximal and distal nerve stumps each with 3–4 stitches of 9–0 nylon sutures. The muscle and skin were then closed using 5–0 polyglactin and 4–0 nylon sutures, respectively. All animals were monitored postoperatively for signs of infection and distress. No auto mutilation or limb contractures were observed postoperatively among the operated animals.

### Isoflurane conditioning

Animals of experimental groups (Group II, III, and IV) were treated with 1-h of 2% isoflurane at 1-h post-surgery and repeated daily for the following 2 days (Group III, total 3 treatments) or 5 days (Group IV, total 6 treatments), respectively, using the custom-made flexi glass chamber (Length – 20″, Width – 10″, and Height − 7″). The dose of isoflurane used in this study was based on previous studies where isoflurane was shown to provide significant neuroprotection in several neurological disorders (11–16).

### Functional assessment

#### CMAPs

Compound muscle action potential assessments began with the exposure and isolation of the operated sciatic nerve as described previously (17). An automated functional assessment station (FASt System, Red Rock Laboratories, St. Louis, MO) supplied monophasic electrical stimuli (1 mA) to the proximal sciatic nerve via epineural hook electrodes and recorded CMAPs from the reinnervated TA muscle using differential recording needle electrodes. The positive and negative recording electrodes were inserted into the belly of the TA muscle, spaced around 5 mm apart. The reference electrode was placed away from the TA muscle at the base of the tail. The CMAP signal was amplified 100× using a 2-channel microelectrode amplifier (Model 1800 A-M Systems, Sequim, WA). The maximum values of the CMAPs were recorded.

### Evoked muscle force

Post CMAP assessment, the distal tendon of the EDL was severed and fixed to a 10 N load cell (S100, Strain Measurement Devices, Wallingford, CT) to measure the evoked EDL muscle forces (17). The elicited twitch contractions were first measured using a single pulse, as well as determining the optimal muscle length for isometric force generation by the EDL muscle. Stimulation frequency was then increased gradually from 80 to 200 Hz to record the tetanic muscle forces. Tetanic muscle force measurements were acquired 2 min apart to avoid muscle fatigue. The maximum tetanic force (*F*_o_) was computed using the FASt System and recorded manually. The specific tetanic muscle force (SF_o_) was normalized by dividing *F*_o_ by the physiological cross-sectional area (PCSA) of the EDL muscle:


$$SF_{o}=F_{o}/PSCA,$$

where the PSCA was calculated by


$$PSCA=\left(M\times \cos\theta \right)/\left(\rho \times L_{o}\times \frac{L_{f}}{L_{m}}\right).$$

In these equations, *M* represents the muscle mass, cosθ represents the angle of pennation of the EDL muscle, ρ represents the density of mammalian skeletal muscle, $L_{o}$ represents the optimal muscle length, and $L_{f}/L_{m}$ represents the ratio of fiber length to muscle length in rat EDL muscle (18).

### Muscle mass

Following the functional endpoint assessments, animals were euthanized by administration of intracardiac sodium pentobarbital. EDL muscles from both the operated and the unoperated side were harvested and weighed immediately. The relative degree of muscle atrophy was quantified using the wet muscle mass ratio between the operated and the unoperated side.

### Histological analysis

The sciatic nerves on the operated side were harvested after animal euthanasia. The nerves were fixed by 3% glutaraldehyde in 0.1 M phosphate solution (PS) for at least 24 h at 4°C. The nerves then underwent osmication overnight, followed by dehydration with 1% osmium tetroxide (Fisher Scientific, Waltham, MA) and gradient ethanol/propylene oxide solutions (Sigma-Aldrich, St. Louis, MO), respectively. The nerves were then embedded into epoxy blocks [Araldite(R) M/hardener DDSA, Sigma-Aldrich, St. Louis, MO]. The nerve embeddings of 5-mm proximal to the trifurcation were sectioned, and stained by 0.5% of toluidine blue (Sigma-Aldrich, St. Louis, MO) for 90s. Nerve sections were then observed under 40, 100, and 1,000× to investigate the axon numbers and structures using Clemex software (Clemex, Fenton, MI).

### Statistical analysis

Statistical analysis was conducted using Origin v8.5 (Origin, Northampton, MA) and GraphPad Prism 8 (GraphPad Software, Boston, MA). All results are reported as mean ± SD. Bartlett test and Kolmogorov–Smirnov test were applied to examine the homogeneity of variances and for normal distribution of the data. Differences between groups were calculated by using a one-way ANOVA followed by Tukey’s HSD multiple comparison test for normally distributed data sets and Kruskal–Wallis followed by Dunn’s multiple comparison test was applied for the data sets which were not normally distributed. The results of statistical test were reported by *p*-value between groups with a significance level of *p < 0.05.

## Results

### Compound muscle action potential (CMAPs)

**Figure 2:**
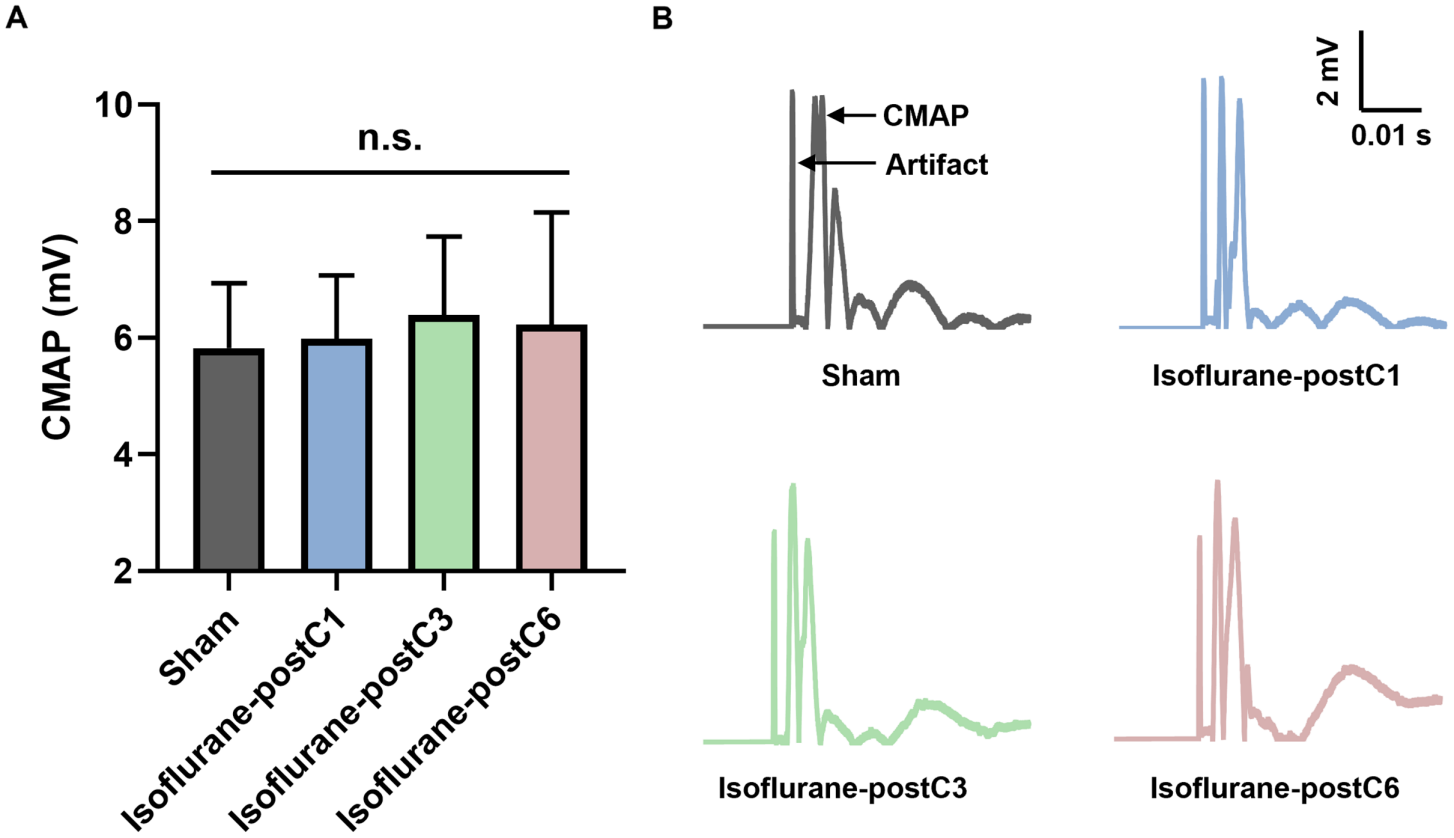
Compound muscle action potentials (CMAPs) after isoflurane conditioning. Amplitude of CMAPs evoked in the tibialis anterior (TA) muscle following 0 Hz stimulation of the sciatic nerve at 12 weeks post-surgery. Data are represented as mean ± SD. **(A)** CMAPs, *p* > 0.05, Sham vs. Isoflurane-postC1, Isoflurane-postC3, Isoflurane-postC6 by ANOVA with Tukey multiple comparisons test; n.s., no significant differences. **(B)** Representative CMAP curves of each group. *N* = 8 individual animals per group. Gray, Sham; blue, isoflurane-postC1; green, isoflurane-postC3; red, isoflurane-postC6.

The CMAPs in reinnervated TA muscle at 12-week post-surgery for each group are showed in Figure 2. Compared to Group I (sham, 5.83 ± 1.11 mV), all isoflurane treated groups demonstrate a trend of higher CMAP values. Although not significant, Group III (6.39 ± 1.35 mV) measured the higher CMAPs than Group II (5.98 ± 1.09 mV) and IV (6.22 ± 1.92 mV).

### Evoked muscle force

**Figure 3:**
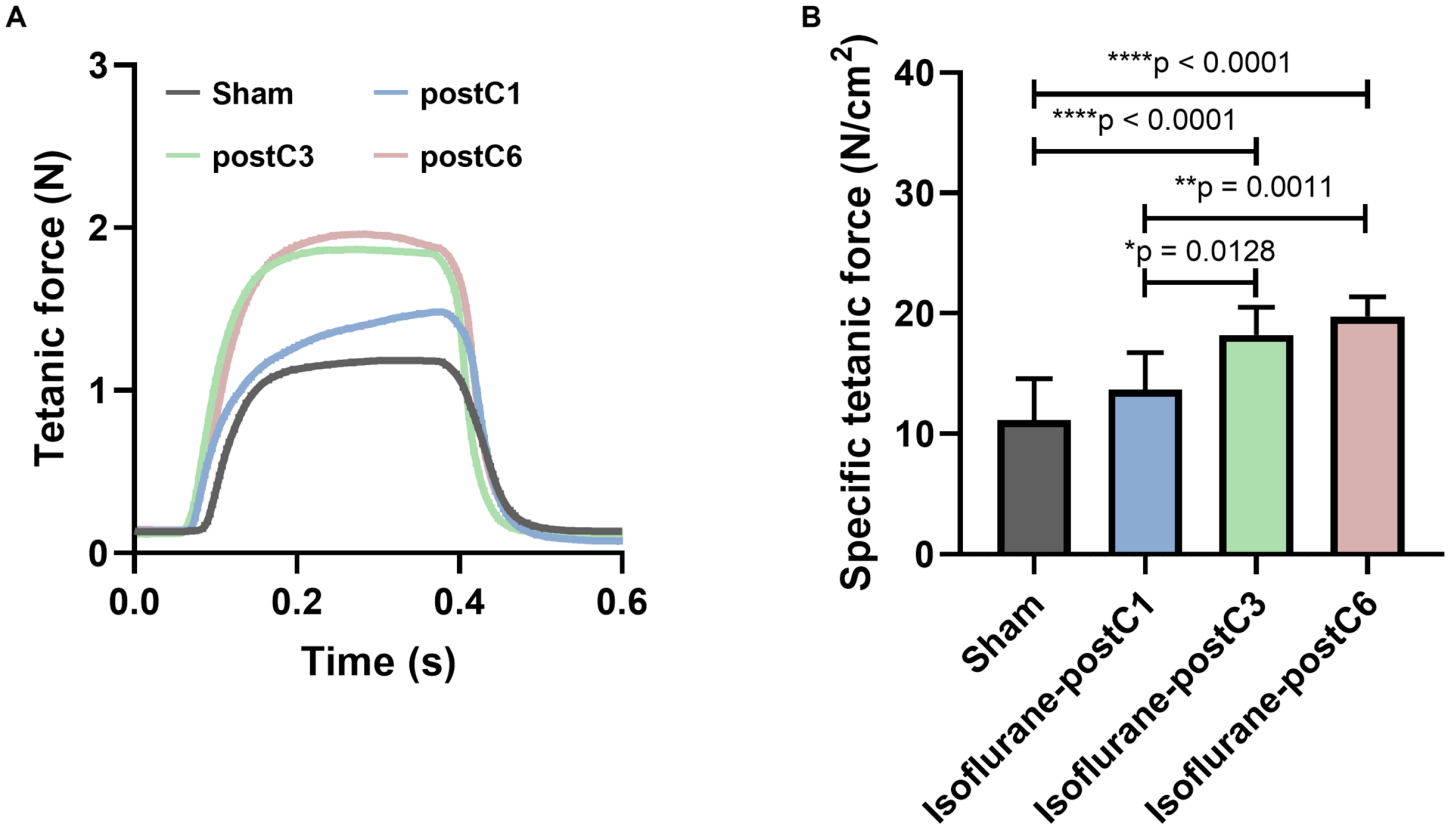
Isoflurane conditioning increased muscle force after PNI and repair. Evoked muscle forces were measured from *extensor digitorum longus* (EDL) after stimulation of the sciatic nerve at 12 weeks post-surgery. Data are represented as mean ± SD. **(A)** Representative tetanic force curves; **(B)** specific tetanic force, *p < 0.05, Sham (11.14 ± 3.46 N/cm2) vs. isoflurane-postC3 (18.23 ± 2.28 N/cm2), isoflurane-postC6 (19.74 ± 1.63 N/cm2); isoflurane-postC1 (13.70 ± 3.03 N/cm2) vs. isoflurane-postC3 (18.23 ± 2.28 N/cm2), isoflurane-postC6 (19.74 ± 1.63 N/cm2); by ANOVA with Tukey multiple comparisons test. *p < 0.05, **p < 0.01, ****p < 0.0001. *N* = 8 individual animals per group. Gray, Sham; blue, isoflurane-postC1; green, isoflurane-postC3; red, isoflurane-postC6.

To illustrate the functional recovery of muscle, the evoked tetanic and specific tetanic force of EDL muscles were measured and analyzed. As shown in Figure 3, repetitive isoflurane conditioning (groups III and IV), resulted in higher muscle force as compared to the sham and single isoflurane conditioning groups (groups I and II). For evoked tetanic and specific tetanic forces, animals in group III and IV showed a significant increase in compared to other two groups I and II, and no significant difference was noted between groups III and IV (Figures 3A,B) (Tetanic forces: Group 1–1.04 ± 0.41 N, Group 2–1.22 ± 0.27 N, Group 3–1.78 ± 0.31, and Group 4–1.83 ± 0.40 N; Specific tetanic forces: Group 1–11.14 ± 3.46 N/cm^2^, Group 2–13.70 ± 3.03 N/cm^2^, Group 3–18.23 ± 2.28 N/cm^2^, and Group 4–19.74 ± 1.63 N/cm^2^).

### Muscle mass

**Figure 4:**
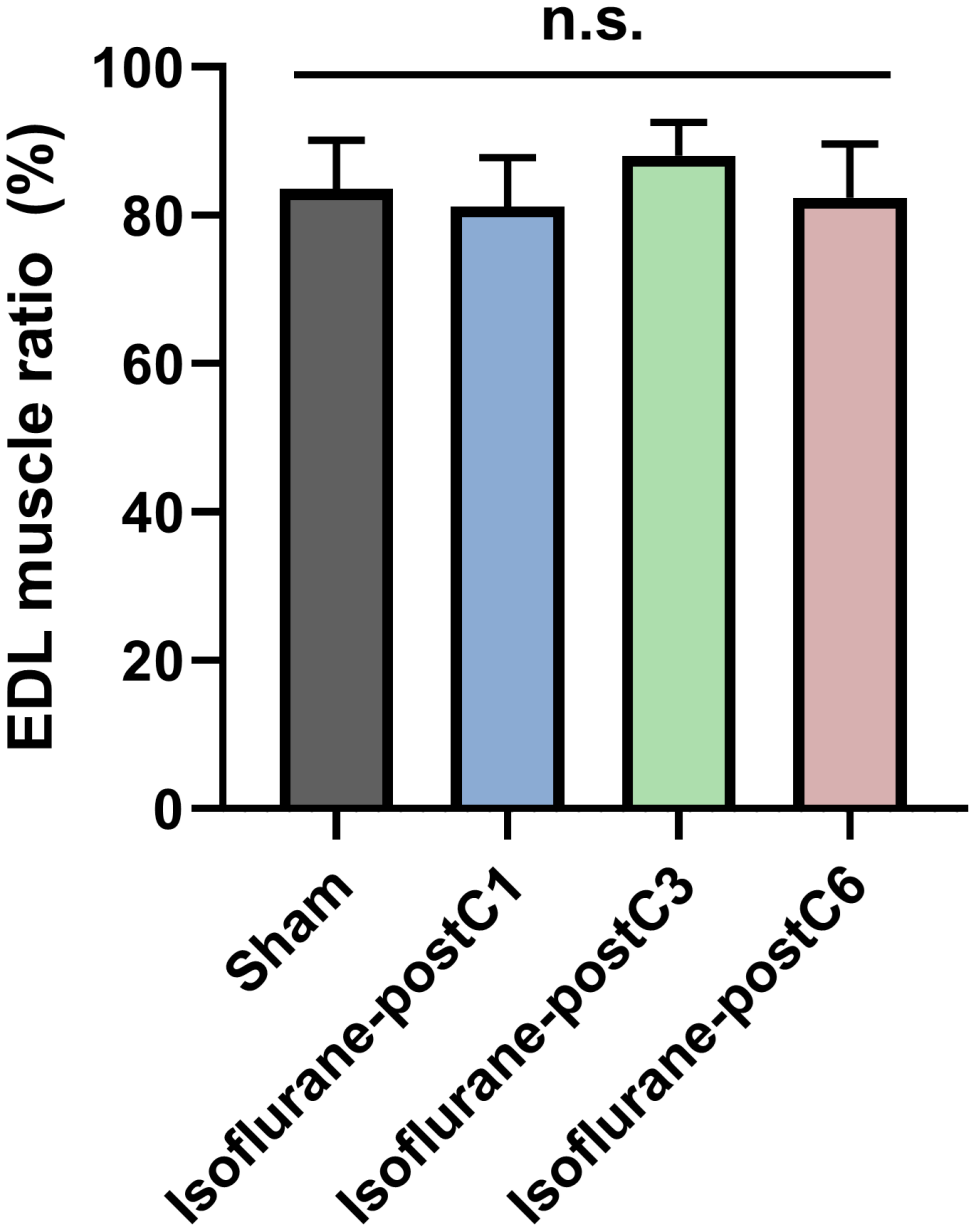
Muscle mass after isoflurane conditioning. Wet muscle mass ratio was measured from extensor digitorum longus (EDL) from both the operated and unoperated side at 12 weeks post-surgery. Data are represented as mean ± SD. n.s., no significant differences. *p* > 0.05, Sham vs. isoflurane-postC1, isoflurane-postC3, isoflurane-postC6, by ANOVA with Tukey multiple comparisons test. *N* = 8 individual animals per group. Gray, Sham; blue, isoflurane-postC1; green, isoflurane-postC3; red, isoflurane-postC6.

Wet muscle mass ratio measured in the EDL on the operated side showed more than 80% recovery in all groups compared to the unoperated side (Figure 4). Though the EDL muscle mass ratio was higher in Group III (88.04 ± 4.52%), it was not significant compared to other three groups (Group I – 83.56 ± 6.57%, Group II – 81.19 ± 6.63%, and Group IV – 82.35 ± 7.25%).

### Histomorphometric analysis of axonal regeneration

**Figure 5:**
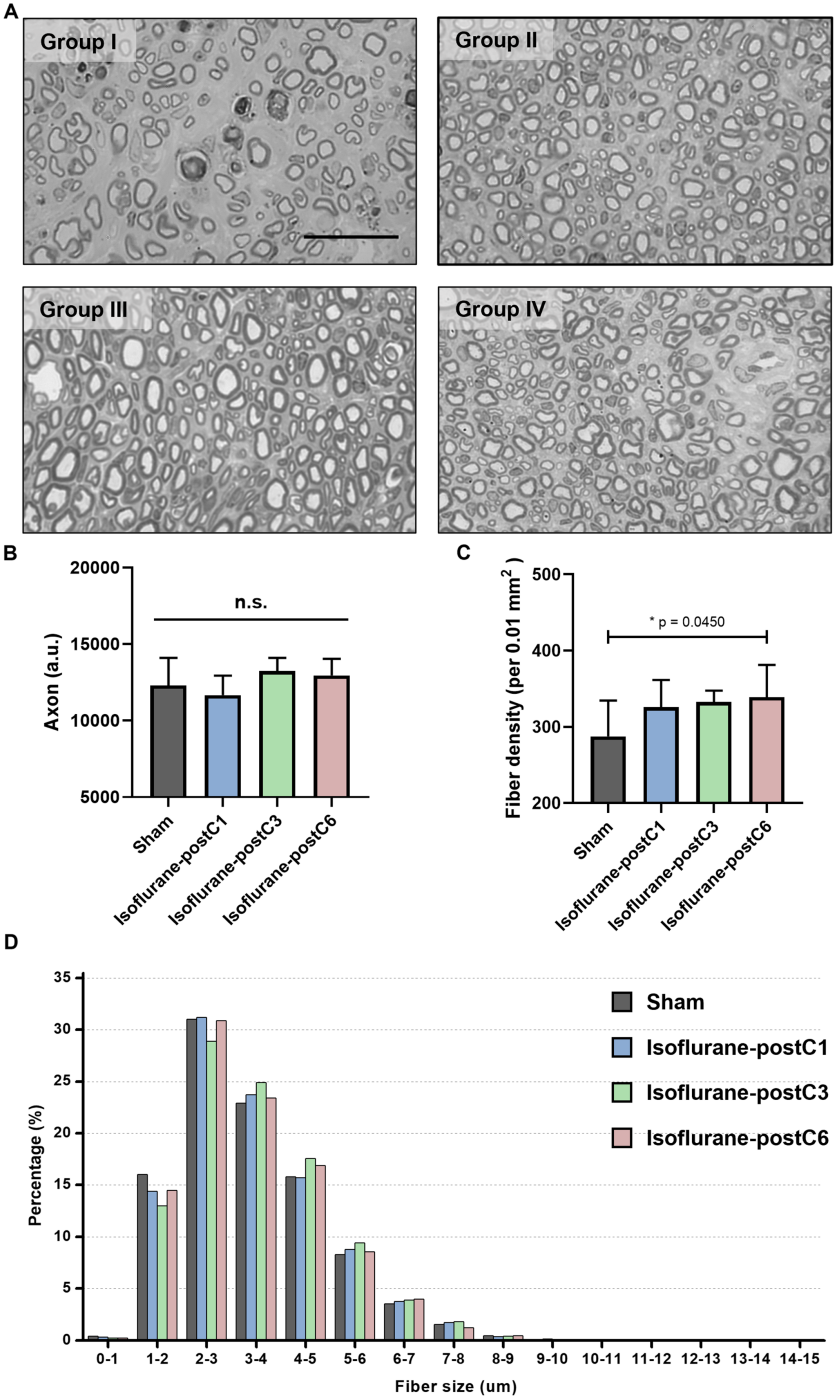
Repetitive isoflurane conditioning promotes axonal regeneration. Axonal counts from the sciatic nerve sections at 12 weeks post-surgery. Data are represented as mean ± SD. n.s., no significant differences. **(A)** Representative microscopic images of the sham and isoflurane conditioning groups. Scale bar, 50 μm. **(B)** Axonal count, *p* > 0.05, Sham vs. isoflurane-postC1, isoflurane-postC3, isoflurane-postC6. **(C)** Fiber density, *p < 0.05, Sham (288 ± 47 per 0.01 mm^2^), vs. isoflurane-postC6 (339 ± 42 per 0.01 mm^2^), by ANOVA with Tukey multiple comparisons test. **(D)** Distributions of fiber sizes in the sham and the different isoflurane conditioning groups. *N* = 8 individual animals per group; *n* = 6 random fields per animal. Gray, Sham; blue, isoflurane-postC1; green, isoflurane-postC3; red, isoflurane-postC6.

**Figure 6:**
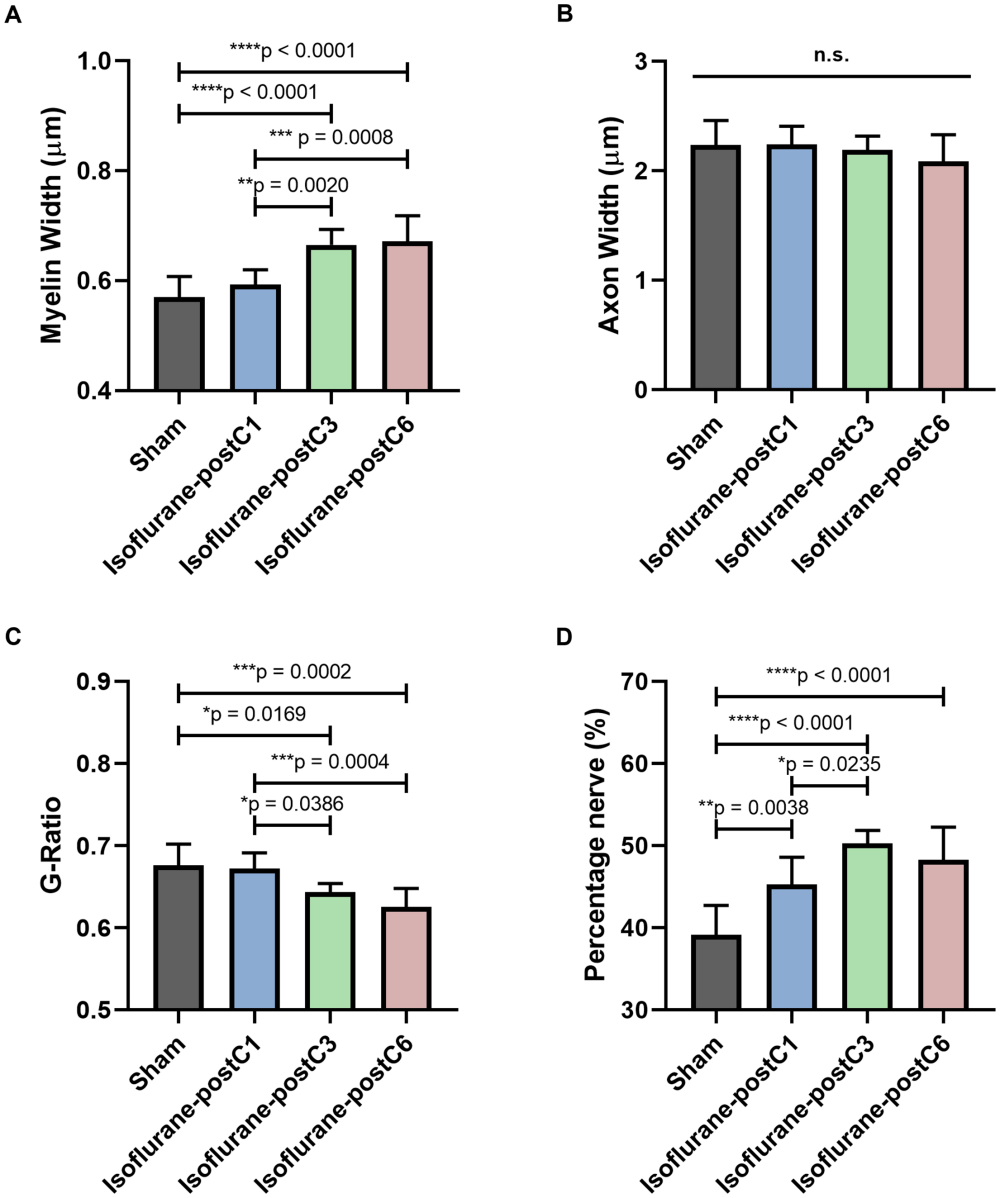
Repetitive isoflurane conditioning improved remyelination. Microscopic analysis of axonal structure from the sciatic nerve sections at 12 weeks post-surgery. Data are represented as mean ± SD. **(A)** Myelin width, *p < 0.05, Sham (0.57 ± 0.04 μm), vs. isoflurane-postC3 (0.67 ± 0.03 μm), isoflurane-postC6 (0.67 ± 0.05 μm); isoflurane-postC1 (0.59 ± 0.03 μm), vs. isoflurane-postC3 (0.67 ± 0.03 μm), isoflurane-postC6 (0.67 ± 0.05 μm). **(B)** Axon width, *p* > 0.05, Sham vs. isoflurane-postC1, isoflurane-postC3, isoflurane-postC6; n.s., no significant differences. **(C)** G-ratio, *p < 0.05, Sham (0.68 ± 0.03), vs. Isoflurane-postC3 (0.64 ± 0.01), isoflurane-postC6 (0.63 ± 0.02); isoflurane-postC1 (0.67 ± 0.02), vs. isoflurane-postC3 (0.64 ± 0.01), isoflurane-postC6 (0.63 ± 0.02). **(D)** Percentage nerve, *p < 0.05, Sham (39.15 ± 3.56%), vs. isoflurane-postC1 (45.29 ± 3.30%), isoflurane-postC3 (50.24 ± 1.59%), isoflurane-postC6 (48.27 ± 3.98%); isoflurane-postC1(45.29 ± 3.30%), vs. isoflurane-postC3 (50.24 ± 1.59%), by ANOVA with Tukey multiple comparisons test (myelin width, G-ratio, and percentage nerve) or Kruskal–Wallis with Dunn’s multiple comparisons test (axonal width). *p < 0.05, **p < 0.01, ****p < 0.0001. *N* = 8 individual animals per group; *n* = 6 random fields per animal. Gray, Sham; blue, isoflurane-postC1; green, isoflurane-postC3; red, isoflurane-postC6.

Figures 5, 6 depict the results of histomorphometric analysis of axonal regeneration. At 12-week post-surgery, all the four groups showed robust axonal regeneration with numerous remyelinated axons and mature nerve architecture (Figure 5A). The total number of axons in Group III (13,245 ± 864) was the highest compared to other groups (Group I – 12307 ± 1793, Group II – 11671 ± 1,269, and Group IV – 12959 ± 1,080) but no statistical significance was observed between the groups (Figure 5B). The density of nerve fibers was increased in all the isoflurane treated groups compared to the sham group, and a significant difference was noted between Group I (288 ± 47 per 0.01 mm^2^), and Group IV (339 ± 42 per 0.01 mm^2^) (Figure 5C). In addition, the percentage of axonal fibers larger than 3 μm was proportionately higher in all the isoflurane treated groups compared to the sham group (Figure 5D).

Figure 6 provides detailed microscopic analysis of the axonal structure. The myelin sheath was significantly thicker in Group III (0.67 ± 0.03 μm) and IV (0.67 ± 0.05 μm) compared to the other two groups (Group I – 0.57 ± 0.04 μm; Group II – 0.59 ± 0.03 μm) (Figure 6A), but no significant difference was identified between the groups with respect to the axonal width (Group 1–2.23 ± 0.23 μm, Group II – 2.24 ± 0.17 μm, Group III – 2.19 ± 0.12 μm, Group IV – 2.09 ± 0.24 μm) (Figure 6B). The G-ratio as defined by the ratio of the inner to outer diameter of a myelinated axon was found to be significantly lower in Group III (0.64 ± 0.01), and Group IV (0.63 ± 0.02), compared to the other two groups (Group I – 0.68 ± 0.03, Group II – 0.67 ± 0.02) (Figure 6C). These findings correlate with the thicker myelin sheath as seen in Groups III and IV. In addition, an increased percentage area of nerve fibers was noticed in all the isoflurane treated groups (Group II, 45.29 ± 3.30%, Group III, 50.24 ± 1.59%, Group IV 48.27 ± 3.98%) compared to sham (Group I – 39.15 ± 3.56%) and among the isoflurane treated groups, group III showed the highest percentage area of nerve fibers. Overall, these findings indicate a significant potential of isoflurane conditioning in promoting nerve regeneration (Figure 6D).

## Discussion

The key finding in our study is that isoflurane conditioning (3- and 6-day groups) promoted axonal and myelin regeneration leading to markedly improved evoked muscle force at 12 weeks post PNI and repair in a murine model. These results suggest a neuroprotective role for isoflurane conditioning in improving functional outcomes after PNI.

There remains a paucity of data on the impact of anesthetics and peripheral nerve regeneration. Recently Cui et al. (19) published their work, demonstrating that continuous infusion of a local anesthetic, ropivacaine improved functional and structural outcomes in a murine sciatic nerve cut and repair model. To the best of our knowledge, this is the first report examining the impact of volatile anesthetics on the functional outcomes after PNI and repair. Here we show that isoflurane conditioning (3 and 6 days) improves functional outcomes at 12-weeks post PNI and repair and no significant differences were noted between the isoflurane (3 and 6 days) groups. Perhaps, isoflurane may have a ceiling effect in improving the functional outcomes. Interestingly, we did not notice a positive impact for a single time isoflurane exposure on the functional outcomes after PNI and repair. This is in contrary to the reports in the literature, where one time exposure of isoflurane has been shown to provide significant neuroprotection in various neurological disorders (11–16, 20). It could be possibly explained by the differences in the time period of end point assessments (short vs. long-term) and the experimental models (CNS vs. PNS) used in these studies.

Residual weakness in the skeletal muscle is a major cause of disability after peripheral motor nerve transection and repair. Our electrophysiological measurements indicate that isoflurane conditioning (3- and 6-day groups) result in a marked increase in specific tetanic force, a sign of muscle reinnervation and a non-significant increase in twitch force and amplitude as measured by CMAPs. Absence of significant differences in the CMAPs may be linked to the relatively smaller sample size, differences in the placement of electrodes during measurements, and possibly due to the inter-individual variations in the axonal regeneration in the animals. Our histomorphometric analysis show that isoflurane conditioning (3- and 6-day groups), significantly increases the conduction velocity of the injured sciatic nerve, which was represented by increase in myelin width, decrease in G-ratio, increased percentage of nerve fibers, and a higher proportion of larger diameter axonal fibers (>3 μm). Though the axonal count was higher in isoflurane conditioning groups (3- and 6-days), it did not reach a statistical significance compared to the sham group. The lack of significance in the axonal count is most likely due to the limited sample size, and investigation of a larger sample size in the future may identify the potential differences.

Interestingly, previous electrophysiological studies have shown that the amplitude of the injured nerve is directly proportional to the axonal regeneration and the conduction velocity of the nerve is related to the remyelination of the axons (21). In our current study, isoflurane conditioning (3- and 6-day groups), resulted in significant myelin reformation compared to the axonal regrowth, suggesting a greater impact of isoflurane conditioning on the conduction velocity of the injured nerve. A recent study exploring the genomic changes induced by isoflurane conditioning indicated that isoflurane significantly upregulated expression of genes such as Mtor, Akt1, and Atrn which are shown to be the major regulators of myelin regeneration (22–24). Though our current study did not directly explore the molecular mechanisms of isoflurane conditioning induced improvement in functional outcomes after PNI and repair, it is possible that these genes may be involved in the underlying neuroprotection and this has to be confirmed in future studies.

Limitations and future directions of the study: (1) Only male animals were utilized for the study and future studies should include female animals; (2) impact of isoflurane conditioning on other PNI models such as sciatic crush nerve injury and isograft/allograft models are not studied; (3) molecular mechanisms underlying isoflurane conditioning induced neuroprotection is not studied; (4) the isoflurane dose utilized in the current study is not a usual clinical dose and hence the therapeutic window for isoflurane conditioning should be examined in future studies; (5) the differential impact of other commonly used general anesthetics (sevoflurane, desflurane, and propofol) on the functional outcomes after PNI have to be explored in future studies; (6) future studies should incorporate behavioral analysis to strengthen the translational aspect of these findings; and (7) finally it is important to note that our current sample size may be limited in power to detect the potential differences in all the assessed outcomes and hence a formal power analysis is warranted for the future studies.

## Conclusion

Overall, our findings suggest that isoflurane conditioning may improve long-term functional recovery after PNI and repair by promoting axonal and myelin regeneration. Further studies exploring the molecular underpinnings of isoflurane conditioning induced neuroprotection after PNI are necessary to identify novel therapeutic targets for improving patient outcomes.

## Data Availability

The raw data supporting the conclusions of this article will be made available by the authors, without undue reservation.
